# Gesture Classification in Electromyography Signals for Real-Time Prosthetic Hand Control Using a Convolutional Neural Network-Enhanced Channel Attention Model

**DOI:** 10.3390/bioengineering10111324

**Published:** 2023-11-16

**Authors:** Guangjie Yu, Ziting Deng, Zhenchen Bao, Yue Zhang, Bingwei He

**Affiliations:** 1 College of Mechanical Engineering and Automation, Fuzhou University, Fuzhou 350108, China; 210227009@fzu.edu.cn (G.Y.); 220227058@fzu.edu.cn (Z.D.); 230220047@fzu.edu.cn (Z.B.); 2 Fujian Engineering Research Center of Joint Intelligent Medical Engineering, Fuzhou 350108, China

**Keywords:** sEMG gesture recognition, deep learning, channel attention mechanism, real-time control

## Abstract

Accurate and real-time gesture recognition is required for the autonomous operation of prosthetic hand devices. This study employs a convolutional neural network-enhanced channel attention (CNN-ECA) model to provide a unique approach for surface electromyography (sEMG) gesture recognition. The introduction of the ECA module improves the model’s capacity to extract features and focus on critical information in the sEMG data, thus simultaneously equipping the sEMG-controlled prosthetic hand systems with the characteristics of accurate gesture detection and real-time control. Furthermore, we suggest a preprocessing strategy for extracting envelope signals that incorporates Butterworth low-pass filtering and the fast Hilbert transform (FHT), which can successfully reduce noise interference and capture essential physiological information. Finally, the majority voting window technique is adopted to enhance the prediction results, further improving the accuracy and stability of the model. Overall, our multi-layered convolutional neural network model, in conjunction with envelope signal extraction and attention mechanisms, offers a promising and innovative approach for real-time control systems in prosthetic hands, allowing for precise fine motor actions.

## 1. Introduction

Upper limb amputees face challenges with activities of daily living and require prosthetic hands to restore their grasping ability. Prosthetic hands can not only improve the physical and psychological quality of life of amputees but also prevent upper limb muscle atrophy and relieve neuralgia [1]. Existing commercial prosthetics often have issues such as high failure rates, limited flexibility, and deficiencies in user–machine interaction. To address these issues, surface electromyography (sEMG) gesture recognition has been implemented into prosthetic hands, which has become one of the most promising technologies for smart prosthetics [2]. The sEMG gesture recognition is a non-intrusive technique that can accurately decode intended hand movements by measuring and analyzing electrical activities generated by muscular contractions and relaxations near the skin’s surface [3], thus enabling the wearer to autonomously control myoelectric prosthetics [4]. However, to achieve a natural and stable autonomous control of myoelectric prosthetic hands to perform various fine hand movements such as grasping, pinching, and fingering, appropriate signal processing methods and the resulting fast, accurate, and reliable gesture recognition are crucial.

Traditional signal preprocessing techniques of bandpass filtering, root mean square (RMS) feature extraction, and frequency domain feature extraction have been extensively employed in processing sEMG signals, among which bandpass filters are widely used to remove noise and interference from sEMG signals, thereby improving signal quality [5]. Meanwhile, RMS feature extraction aims to obtain amplitude information from sEMG signals, and then the obtained amplitude information is used as features for gesture classification [6] or as a fixed threshold for identifying the initiation and cessation of dynamic activities. Frequency domain feature extraction methods transform the signals into the frequency domain to extract spectral information. These traditional signal processing techniques possess attributes such as low complexity and quick processing time, rendering them advantageous for real-time control, but they exhibit limitations in terms of data processing and generalization capabilities, often necessitating the manual adjustment of model parameters to accommodate new samples. Worse still, these traditional techniques may introduce signal distortion, unintentionally omit vital feature information during the noise reduction process, or fail to adequately capture the precise dynamic changes and temporal relationships inherent in the signals, so they are suitable for distinct gesture classification with small-scale sEMG data [7].

Deep learning techniques have recently garnered significant attention for their ability to automatically extract hierarchical and abstract features from raw data [8]. Notably, convolutional neural networks (CNNs), recurrent neural networks (RNNs), and their variants have been successfully applied to gesture recognition using sEMG signals. These deep learning models excel in capturing complex temporal and spatial dependencies, resulting in more accurate, fine, and robust gesture classification [9]. Based on a large-scale sample set including 30 gestures from 12 able-bodied subjects, Shi et al. proposed a CNN architecture and proved its potential in sEMG pattern recognition after multiple training and optimization [10]. Sun et al. [11] employed a long short-term memory (LSTM) model to identify the motor intention of neurobots based on high-density sEMG data, and the model achieved a peak accuracy of 83.33%. Likewise, the dataset was also large-scale, consisting of recordings from 19 participants encompassing 65 different gesture classes, and a significant amount of time was devoted to training. Chen et al. [12] proposed an effective transfer learning strategy using a CNN-LSTM model for sEMG-based gesture recognition. They selected a dataset consisting of 30 hand gestures involving various states of finger joints, elbow joints, and wrist joints. After about 800 epochs of training and with all networks converged, over 90% recognition accuracy was achieved on the training dataset. Although fine gesture recognition with high accuracy was achieved, the network structures of the above models were complex and required large-scale datasets with many computational resources in the training phase, thus requiring a long training and computational time. Additionally, these models may face challenges in accuracy degradation during practical testing, possibly due to weak generalization ability or interference from real-world environments.

In the past five years, attention mechanisms have been increasingly integrated into deep learning architectures to enhance the discriminative power and robustness of gesture recognition models by selectively attending to informative regions or channels of sEMG signals [13]. Hu et al. [14] proposed a gesture recognition model called “MIC-Attention-LSTM”, which used correlation numbers to reduce time-domain features, used the maximal information coefficient (MIC) to select the best feature set, and utilized LSTM and Attention-LSTM to create the final model. The MIC-Attention-LSTM model achieved a higher classification accuracy of 87.27% compared to the LSTM model with the same architecture, highlighting the effectiveness of the attention mechanism algorithm in enhancing LSTM classification accuracy. Similarly, Lv et al. [15] proposed a remote hand gesture recognition system based on a multi-attention deep learning framework with multi-view, which utilized a sparse complex-valued neural network to automatically process channel selectively and obtain the final output by summing the rescaled transformation output and original data. The proposed framework was validated on the myo dataset, myoUp dataset, and ninapro DB5, achieving improved accuracy rates of 0.46%, 18.88%, and 7% compared to previous works, respectively. Because attention mechanisms focus more on the relationships of local features, they are limited in capturing cross-channel interactions and often show limited adaptability to different gesture recognition tasks and datasets. Therefore, additional hyperparameter tuning and model structure design are often required to make these attention mechanisms better adapted to specific application scenarios. However, this process can be time-consuming and computationally intensive, which increases the complexity of the models and may have the opposite effect on improving the real-time performance of the recognition system.

To solve the aforementioned challenges, this study proposes a unique and low-complexity model for gesture recognition termed CNN-ECA, which combines a CNN with an enhanced channel attention (ECA) mechanism to improve its discriminative power. To progressively expand the receptive field size, the model incorporates stacked convolutional layers with varying kernel sizes. On this basis, the addition of the ECA mechanism can extract the dependency relationships between the sEMG data of different channels via cross-channel interactions, and adaptively obtain the feature channel weights via attention weighting, effectively focusing on relevant features and improving the model’s robustness in capturing essential information from gesture sEMG signals. In addition, to make the signal input into the model more concentrated and stable in both the time domain and frequency domain, a preprocessing strategy for extracting envelope signals using the fast Hilbert transform (FHT) is developed. The combination of these techniques yields a powerful and efficient model for gesture recognition, allowing for the real-time control of prosthetic hands and potentially beneficial applications involving accurate and dependable sEMG signals.

## 2. Materials and Methods

This section presents the methodology employed in this study for gesture recognition based on sEMG signals. It encompasses the description of the dataset, data preprocessing, overlapped sliding window segmentation, CNN-ECA architecture, and the design of the prosthetic hand system.

### 2.1. Dataset Description

#### 2.1.1. sEMG Acquisition Hardware

The dataset used in this study plays a vital role in training, evaluating, and validating sEMG-based gesture recognition systems. To ensure optimal signal handling, it is essential to select reliable equipment to collect sEMG signals. gForcePro+ armband from OYMotion Technologies (Shanghai, China) with an 8-channel high-sensitivity myoelectric sensor was employed in this work, and each myoelectric sensor consists of three differential dry electrodes and an electronic component. The myoelectric sensor collects sEMG signals through the electrode’s contact with the skin, and the internal electronic component performs amplification, filtering, sampling, and digitization. Since sEMG signals in the frequency range of 20–550 Hz contain the most relevant information for motion recognition [16], the sampling frequency of the gForcePro+ armband was set as 1000 Hz to allow us to preserve the information to the greatest extent and ensure that the dataset is suitable for our study’s objectives.

#### 2.1.2. Hand Gestures

**Figure 1:**
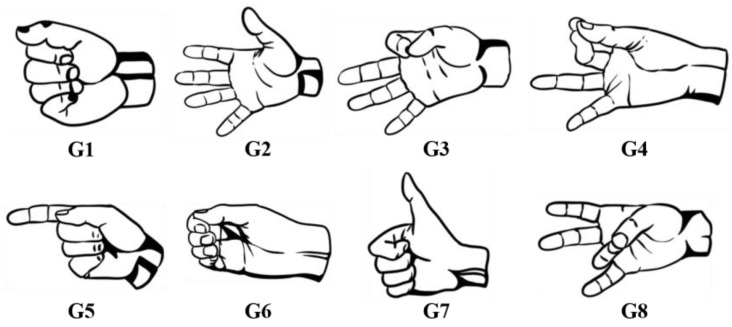
Hand gesture examples.

Eight gestures (G1~G8) that are commonly used in daily life and can be performed by prosthetic hands are involved in the experiment. As shown in Figure 1, these gestures cover a wide range of functions and are described below:

G1: Fist—This gesture involves making a fist, which is used to grab or hold an object.

G2: Open—In this gesture, the hand opens up, allowing the release of the held object.

G3: Two-Finger Pinch—The index finger and thumb come together to pinch, which is used to hold small or flat objects.

G4: Three-Finger Pinch—In this gesture, the thumb pinches the index finger and the middle finger, used for holding round objects.

G5: Pointing—The index finger is extended to point towards a certain direction or target.

G6: Hook—The four fingers are bent, resembling a hook, and used to pick up objects like bags.

G7: Thumbs Up—This gesture involves giving a thumbs-up, expressing approval, encouraging, or signaling positivity.

G8: Ring Finger Grasp—In this gesture, the thumb is brought towards the ring finger, used for grabbing small objects or rotating bottle caps, among other things.

#### 2.1.3. Acquisition Protocol

**Figure 2:**
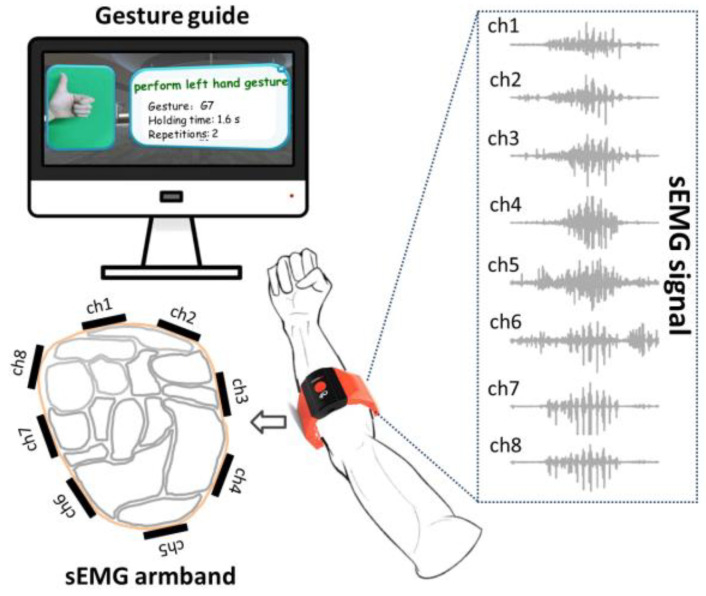
Diagram of the acquisition experiment.

We recruited 10 participants, 7 males and 3 females, with ages of 30 ± 5 years old. The experiment protocol was reviewed and approved by the ethics committee of Fujian Provincial Hospital (approval number: K2022-09-015). None of the participants have suffered from upper extremity muscle pain or orthopedic diseases in the past year, and they were all informed about the content and purpose of the experiment. Before the experiment, the participants sat on the side of an experimental table and kept their right forearm resting on the table. Then, the myoelectric armband was worn on the middle of the forearm, ensuring that the internal metal electrode was in close contact with the skin. All participants were asked to practice each gesture until they were able to perform the task as required by the experiment. During data collection, they were asked to complete each gesture by imitating a video of gesture instructions displayed on a computer screen (Figure 2). To capture natural variations in muscle activation patterns, the acquisition time for each gesture was set to one minute, during which each gesture was repeated approximately 15–20 times. To avoid muscle fatigue, the participants were given a five-minute rest after completing each gesture.

### 2.2. Data Preprocessing

Previous studies on gesture recognition demonstrated that extracting the envelope of the raw sEMG signals could provide valuable insights into muscle activity [17]. The envelope of the sEMG signals is a smoothed curve obtained by processing the raw signals, which simplifies data representation, reduces noise, expresses motion actions, and plays a crucial role in the classification of sEMG signals. Several methods are commonly used for envelope extraction, such as moving average, envelope detection, Hilbert transform, and wavelet transforms [18]. In this study, considering the performance requirements of real-time control and fine control for the use of prosthetic hands, the FHT is employed to extract the envelope of the sEMG signals.

**Figure 3:**
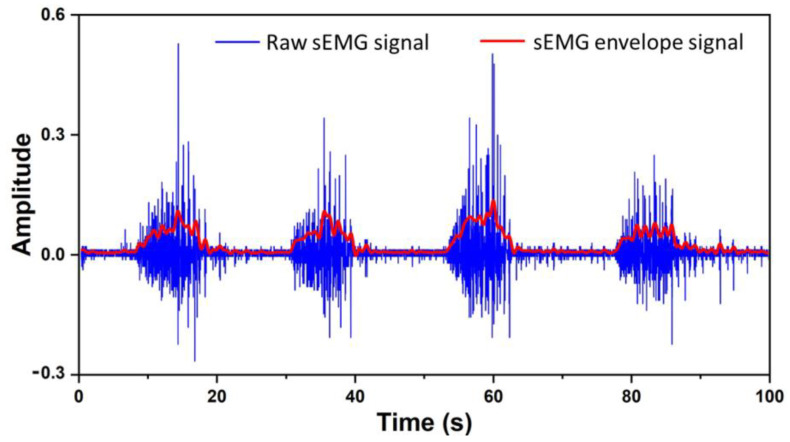
Envelope signal extraction.

The FHT is an efficient signal processing technique used for extracting analytic envelopes from time-domain signals [19]. During its computational process, the input time-domain signal is initially transformed through a Fourier transform to be mapped to the signal into the frequency domain. The FHT then performs phase adjustments according to the properties of the frequency domain signal, resulting in the imaginary part of the transformed signal corresponding to the original signal’s envelope, while the real part corresponds to the signal’s instantaneous phase. The key advantage of the FHT lies in its computational efficiency, which enables rapid and effective envelope extraction, thereby providing robust support for the control and recognition of sEMG signals. The extracted envelope signal is illustrated in Figure 3.

### 2.3. Overlapped Sliding Window Segmentation

**Figure 4:**
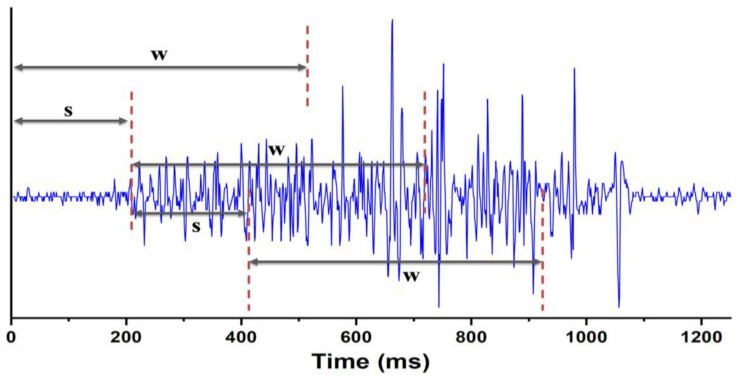
Window segmentation of the sEMG signal.

We employed an overlapped sliding window technique to segment the previously extracted envelope dataset of muscle sEMG signals. This approach allows us to control the overlap between data windows by adjusting the window size and step size, and decomposing the signals into muscle activity segments for feature extraction [20]. The window size (w) determines each window’s duration. The step size (s) defines the time interval between windows, controlling their sliding speed. A smaller step size leads to increased overlap and provides more data for training and evaluation. In this approach, adjacent windows partially overlap, as shown in Figure 4. This sliding window-based segmentation effectively addresses the temporal characteristics of muscle sEMG signals, enhancing the gesture recognition system’s accuracy and robustness [21]. Using this technique, we extract relevant temporal information from sEMG signals, improving the system’s ability to identify and recognize different gestures.

We initially collected datasets from 10 participants, with each gesture lasting for one minute. Considering the duration of the gestures, real-time system requirements, and efficient data utilization, we set the window size to 300 ms with a step size of 100 ms. This segmentation approach allowed us to effectively extract and analyze relevant information from the dataset. Once the data was segmented, we divided it into training, validation, and test sets. Specifically, we allocated 80% of the dataset for the training set, 10% for the validation set, and the remaining 10% for the test set. This distribution allows us to evaluate the model’s performance on different datasets, ensuring a comprehensive assessment of its capabilities.

### 2.4. CNN-ECA Architecture

Deep learning algorithms offer several advantages for sEMG signal classification and recognition, including fast training and inference speed, suitability for real-time applications, high flexibility, and the ability to automatically extract features without manual intervention [22]. Although sEMG signals are relatively stable during rest, they exhibit rapid changes to reflect muscle activity when gestures are performed. Once the gesture is completed, the changes in the sEMG signals cease, and the signals remain relatively stable, no longer representing a temporal sequence of changes [23]. In the context of classifying and recognizing sEMG signals, it becomes crucial to focus on the signal variations during the gesture rather than the continuous state. As such, the classification model should emphasize capturing the dynamic features of gesture changes rather than modeling long-term sequences. An RNN like LSTM is commonly used for processing long-term temporal dependencies but might not be well suited for short-duration sEMG signals. On the other hand, CNNs excel at automatically learning local features in the temporal sequence, demonstrate faster training speeds than LSTM, and are particularly suitable for handling sEMG signals collected from multiple muscle channels.

**Figure 5:**
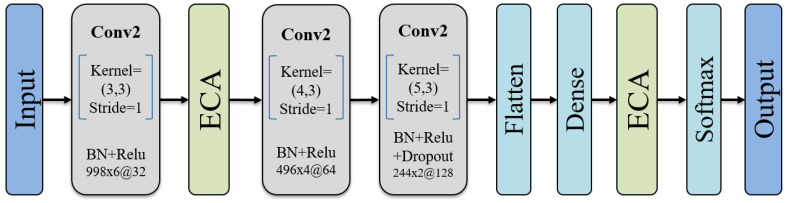
The CNN-ECA structure.

The architecture of the proposed attention-fused CNN model primarily consists of convolutional layers and attention mechanism layers (Figure 5). Specifically, each convolutional layer is followed by a batch of normalization (BN) layer and rectified linear unit (ReLU) activation function to speed up convergence and address the problem of gradient explosion. To handle channel data from different muscle positions, the sizes of the convolutional kernels in the first, second, and third layers are set to (3, 3), (4, 3), and (5, 3), respectively. By sliding these kernels along the temporal and channel dimensions, the network can capture dynamic patterns in the temporal sequence and correlations among different channels simultaneously. Furthermore, the design of stacking kernels of different sizes enables the convolutional layers to gradually increase their receptive field, which is beneficial for capturing more comprehensive features. It is worth noting that the ECA mechanism layer is integrated after the first convolutional layer. This layer can enhance the focus and capture ability of important features through an adaptive weight allocation mechanism, contributing to improved model performance and generalization. At this stage, the model has already attained a wealth of feature expression capabilities; however, there may still be instances of redundant or less relevant features. To introduce a more nuanced emphasis and adjustment within the model’s decision-making layer, another ECA mechanism layer is integrated after the final fully connected layer. The incorporation of the ECA mechanism at this juncture directs the model’s focus towards features that significantly impact the current gesture classification task during decision making, thereby enhancing the model’s ability to distinguish complex EMG signals. Besides, to prevent overfitting, we incorporated dropout layers. Finally, a softmax layer with nodes equal to the number of gesture categories was added to convert the model’s output into a probability distribution for each class, enabling the effective classification of the gestures.

Furthermore, the inclusion of BN layers and dropout layers obviates the necessity to reduce the learning rate, as they can improve the model’s stability and generalization capability, facilitating smoother convergence. To optimize the model’s training process, the ADAM optimizer is adopted, which is a gradient-based optimization algorithm that efficiently updates the model’s parameters to minimize the loss function. For the loss function, we opted for categorical cross-entropy, a widely used metric for multi-class classification problems. This metric quantifies the disparity between the model’s predictions and the actual labels, and can guide the optimization of the model’s parameters [24].

**Figure 6:**
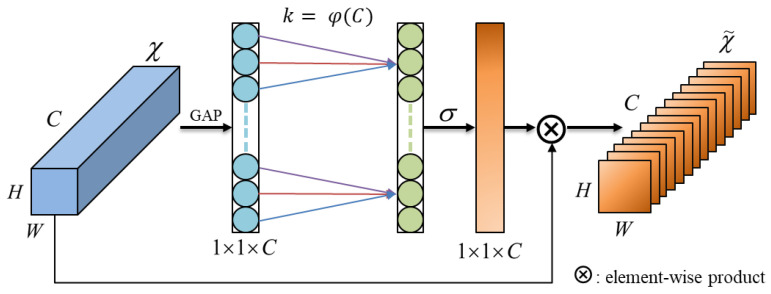
Structure of the ECA module.

The attention mechanism is a machine learning technique that assigns different weights to input features in a neural network. The ECA mechanism utilized in this study is a form of channel attention mechanism, representing an enhanced version of the Squeeze-and-Excitation Networks (SE-Net) [25]. By adaptively weighting the channel dimension of input features, the model can better capture important information in sEMG gesture signals. Its structure is depicted in Figure 6, where the fully connected layer in the original SE-Net is replaced by one-dimensional convolutional kernels, making the model more lightweight. The size of the one-dimensional convolutional kernel is automatically determined based on the number of channels, eliminating the need for manual adjustments. The formula is as follows:

(1) $$K=\varphi \left(C\right)={\left|\frac{{log}_{2}\;c+1}{2}\right|}_{odd}$$

where K represents the kernel size, C represents the number of channels, and ${\left|x\right|}_{odd}$ denotes the nearest odd number to *x*. By adapting the kernel size according to the number of channels, we can fully integrate interactions among specific channels. This technique substantially reduces the model’s complexity while preserving its performance. Moreover, it effectively mitigates the impact of dimensionality reduction on channel attention learning, resulting in notable improvements in the model’s processing speed and accuracy.

### 2.5. Real-Time Prosthetic Hand Control System

**Figure 7:**
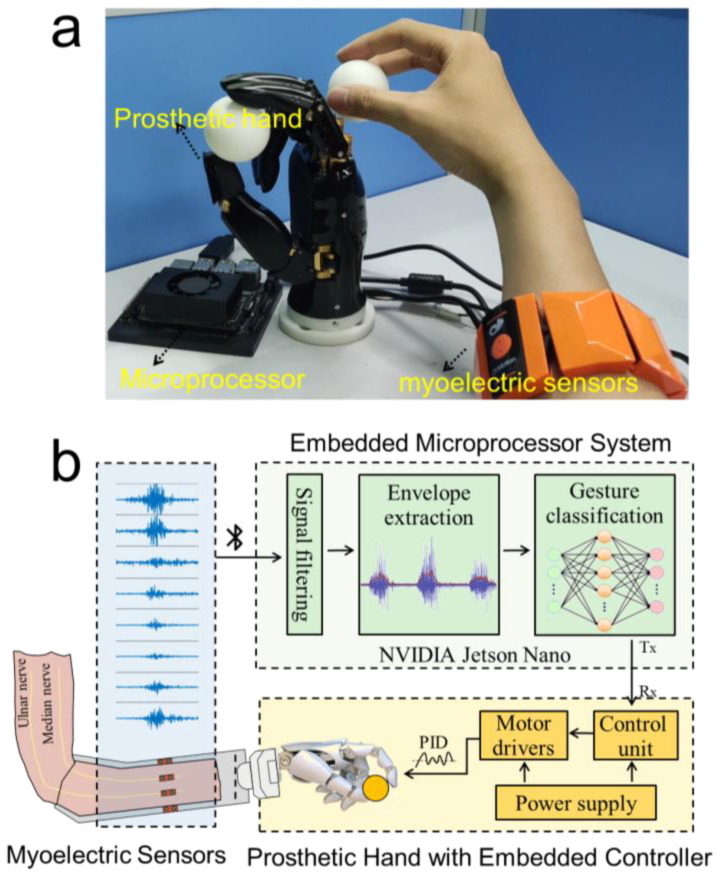
The prosthetic hand control system. (**a**) Control system hardware and (**b**) Control system flowchart.

As shown in Figure 7a, the hardware of the prosthetic hand control system we proposed is composed of three parts: the myoelectric sensor, the prosthetic hand, and the microprocessor. Among these, the myoelectric sensor is responsible for the real-time collection of the sEMG data when the participant performs the designed gesture. The microprocessor receives the sEMG data collected by the sensor in real-time through Bluetooth, processes and classifies the data, infers the participant’s gesture intention, and sends control commands to the prosthetic hand to perform the intended gestures. To improve the real-time performance of the prosthetic hand control system, we carefully selected the system hardware, adjusted its parameters, and designed the microprocessor. Among them, the information on the sEMG sensor has been given above, and then we will focus on describing the relevant information about the prosthetic hand and the microprocessor.

The dexterous prosthetic hand utilized in this study features a 5-finger design equipped with a linkage transmission mechanism. It incorporates two degrees of freedom (DOFs) for the thumb and one DOF for each of the other fingers, amounting to a total of six DOFs for all five fingers. This configuration allows for the independent control of the bending of the thumb, index finger, middle finger, and ring finger, as well as the independent control of the rotation of the thumb. As a result, it can closely simulate human hand movements. The motors and control circuitry of the prosthetic hand are fully integrated within the hand itself, streamlining its appearance and functionality. Moreover, the hand can be conveniently connected to a computer or other embedded systems through a serial communication protocol, enabling seamless communication and control.

To balance the accurate recognition of intention gestures and the real-time control of the prosthetic hand, the requirements of the system for high performance, small device size, and low power consumption were carefully considered when the system was designed, and the NVIDIA Jetson Nano was employed as the embedded microprocessor. This microprocessor offers a high-performance GPU accelerator and a dedicated deep learning inference engine, allowing for fast and real-time inference capabilities [26], which make it particularly suitable for applications such as real-time control. Furthermore, the compact size and low power consumption of the microprocessor make it highly convenient to integrate into the prosthetic hand system for compact assembly and enhanced portability. Running on the Jetson Nano microprocessor is the data preprocessing algorithm and pre-trained CNN-ECA model (Figure 7b), which was developed using the Python programming language by integrating several popular data analysis tools and libraries such as pandas, scipy, loadmat, numpy, and matplotlib. Specifically, the microprocessor received the raw data stream from the myoelectric sensors in real-time through a multithreaded Python program and called the SciPy library to perform signal reprocessing such as filtering and envelope extraction. Then, the reprocessed signals were streamed into the pre-trained CNN-ECA model built on Keras in the TensorFlow library for gesture recognition. The recognition outcomes were converted into control commands and transmitted to the prosthetic hand through the serial port at a baud rate of 115,200. Upon receiving the control commands, the prosthetic hand executed the corresponding gesture through the motor drive module to realize real-time response and control synchronized with the sEMG signals. A threshold value (T) was established based on the envelope signal to prevent the misclassification of motions during muscular relaxation. When the signal exceeded the threshold T, the recognized command was sent to the prosthetic hand, controlling it to do the appropriate action. If the signal dropped below the T threshold, the prosthetic hand stayed in a resting posture. This method ensures accurate and precise control of the prosthetic hand while taking the user’s muscle activity into account.

To ensure the precise and real-time control of the prosthetic hand, we employ the majority vote window technique. This approach collects multiple gesture predictions within a specific time window and selects the final recognized gesture based on the most frequently predicted gesture, following the majority vote principle [27]. For instance, if we set the majority window size to 10, this means that the model’s predictions for the current gesture are considered in conjunction with the preceding and subsequent 10 predictions within the specified time frame. The final prediction is then determined by the majority vote within this window. The majority window size can be adjusted to balance between rapid response and recognition stability, enhancing the reliability of the system. This technique smooths out momentary misclassifications, resulting in the accurate and dependable control of the prosthetic hand in harmony with the user’s muscle activity.

## 3. Results

### 3.1. Ablation Experiment

To validate the rationality and effectiveness of the proposed CNN-ECA network model, it is necessary to design and conduct ablation experiments to assess and understand the importance and contribution of each component in the model, as well as their impact on the model’s performance [28]. A one-dimensional convolutional network without the attention mechanism (Conv1D) serves as the baseline model for the ablation experiments. The following variants were tested: (a) a network with two-dimensional convolutional layers (Conv2D), (b) the addition of the ECA module to the network in (a) (Conv2D+Attention), and (c) the inclusion of preprocessing on top of (b) (i.e., our proposed CNN-ECA). The 1D and 2D convolutional layers maintained consistent kernel sizes in the temporal dimension and retained the same number of kernels and stride parameters.

**Figure 8:**
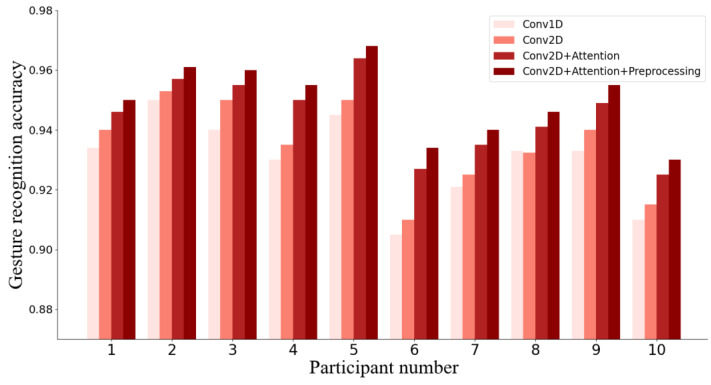
The results of each participant’s ablation experiment.

Figure 8 presents the comparison of accuracy in the ablation experiments at different steps for 10 participants in the collected dataset. As the experiment progresses, the accuracy consistently exhibits an increasing trend, indicating an improvement in performance with more modules. Specifically, with the introduction of the attention mechanism (approach b), the model’s performance significantly improves, highlighting the positive impact of the attention mechanism on the model’s accuracy. Furthermore, by applying our proposed preprocessing method on top of approach b (approach c), the model’s performance is further enhanced. This finding highlights the effectiveness of the preprocessing approach we designed in this study, and demonstrates its autonomous ability not only to enhance the model’s robustness and accuracy but also to further amplify these attributes when integrated with the attention mechanism.

The results presented in Table 1 provide a quantitative summary of the ablation experiments. It is obvious that using Conv2D improves accuracy to 0.89 while decreasing loss to 0.36 in the gesture classification task when compared to Conv1D. This boost in performance can be attributed to the 2D layers’ capacity to capture dynamic patterns in the time series of the sEMG signals as well as the correlations among multiple channels, resulting in a more comprehensive representation of the data. Moreover, the introduction of the ECA module improves the accuracy of the Conv2D model to 0.92 and reduces the loss value to 0.31, because the attention mechanism can improve the model’s ability to capture key information from the data by focusing on important features and channels, and thereby produce more accurate and meaningful predictions. It is worth noting that the above performances were all obtained from the raw data. When the raw data is preprocessed and then the gesture classification task is performed based on Conv2D+Attention, the highest accuracy of 0.96 and the smallest loss of 0.16 are achieved. The above ablation experiments, on the one hand, prove the necessity of data preprocessing, and on the other hand, they demonstrate the advantages of our proposed CNN-ECA model in gesture classification accuracy.

### 3.2. Model Evaluation

In deep learning applications, even when a suitable architecture is found, it is still necessary to search for optimal parameters to improve the performance of the algorithm [29]. In this study, we adopted a systematic approach to fine-tune the model’s hyperparameters. We examined each parameter individually while keeping other parameters constant, so that we could observe the specific impact of each parameter on the model’s learning process. At the end of each test, we retained the hyperparameter values that yielded the best results for subsequent testing.

To evaluate the model’s performance during the training phase, we used the validation set after each training cycle epoch and gained insight into how the model performed during training by calculating accuracy and other metrics. If the model’s accuracy improved, we preserved the weights and biases of the current model. Once the predefined number of epochs was reached, we selected the model with the best performance from the validation set for further testing and halted the training process. This iterative approach guarantees that the best-performing model is chosen after each iteration, allowing for more accurate predictions during subsequent testing phases. Table 2 displays the final set of optimal parameters obtained from this method, illustrating the highest performing configuration for the model.

**Figure 9:**
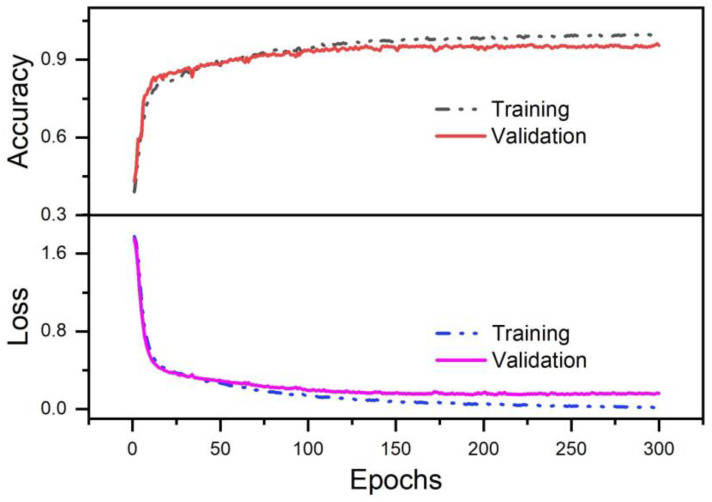
Loss and accuracy graph.

**Figure 10:**
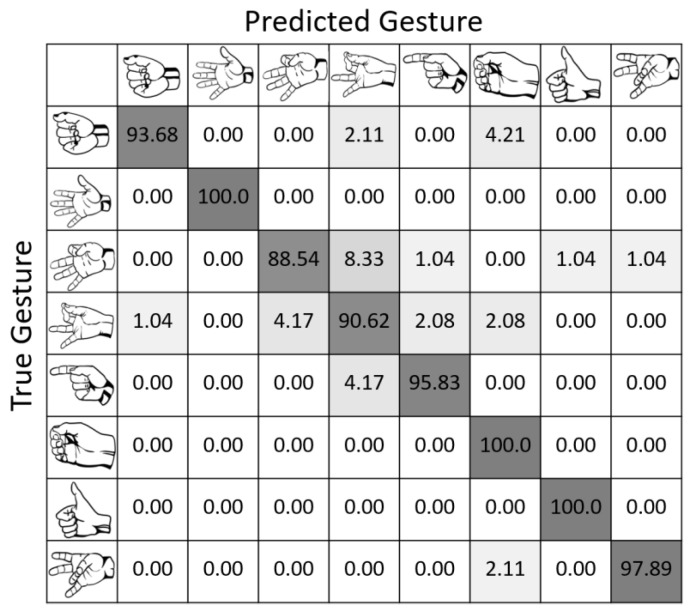
The confusion matrix.

The model evaluation is represented by the accuracy and loss curves and the confusion matrix. Figure 9 demonstrates that our model produced good results, with an accuracy of 99.69% after 300 iterations during the training phase and 95.56% during the testing phase. The close proximity of the two accuracies suggests that there is no overfitting and that the model generalizes effectively to fresh data. Furthermore, the confusion matrix in Figure 10 provides deeper insights into the model’s performance across individual gesture classes. Obviously, our proposed CNN-ECA can perfectly recognize gestures such as “Open”, “Hook”, and “Thumbs Up” with 100% accuracy, while the recognition accuracy for the fine gestures of “Two-Finger Pinch” and “Three-Finger Pinch” is only 88.54% and 90.62%, respectively. This is due to the fact that fine gestures require complicated muscle coordination, which might result in comparable muscle activation patterns or tiny differences that are difficult to discern, thus resulting in occasional misclassifications by the model. Despite its lower accuracy for these fine gestures, the model remains robust across the entire set of gestures. The average accuracy of the model is 95.45%, which demonstrates the reliability and effectiveness of our model in gesture classification.

**Figure 11:**
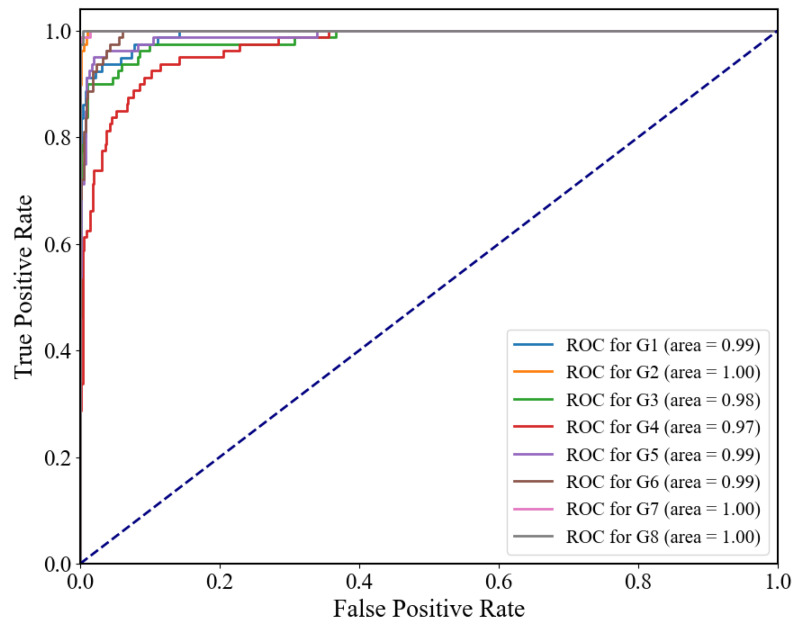
ROC curves for the CNN-ECA model in gesture classification.

In addition to these metrics, we introduced the receiver operating characteristic (ROC) curve in Figure 11, which provides a comprehensive evaluation of the model’s performance across various thresholds. The AUC (area under the curve) of the ROC curve is an additional metric that signifies the model’s ability to distinguish between different classes. A higher AUC value indicates superior discriminative power, implying that our model excels in accurately distinguishing between gestures. A higher AUC value, observed in our model, signifies excellent discrimination between gestures. This suggests that our CNN-ECA model achieves a remarkable balance between sensitivity and specificity, crucial for accurate gesture classification.

### 3.3. Comparison with Other Gesture Recognition Models

We conducted a comprehensive comparison between our proposed CNN-ECA model, classical machine learning models of K-nearest neighbor (KNN) and support vector machine (SVM), as well as deep learning models including LSTM and the current state-of-the-art deep learning model of CNN-LSTM for sEMG gesture recognition to validate the performance of our approach. To ensure consistent and fair comparisons, we split the same dataset into 300 ms windows for all these models, maintaining the same class proportions as the original dataset, and set a fixed random seed to guarantee identical training, test, and validation sets. Throughout the experiments, the learning rate was uniformly set to 0.0001, and training was stopped when the model achieved optimal accuracy. We evaluated the classification model’s performance using common metrics such as accuracy, recall, F1 score, and precision.

The specific experimental results are shown in Table 3, which indicates that classical machine learning approaches have relatively poor performance metrics (i.e., accuracy) in gesture recognition, which are potentially due to limits in feature extraction and model expressiveness. Moreover, the CNN-LSTM model outperformed the LSTM model, providing it with an edge in EMG gesture recognition, because convolutional networks can better capture the correlation between multi-channel sEMG data and gesture motions. Compared with the above gesture recognition models, the introduction of the ECA mechanism enables our proposed CNN-ECA model to effectively extract and utilize relevant features to improve classification performance.

### 3.4. Real-Time Performance Metrics and Results

As a key indicator for evaluating the quality of EMG-controlled prosthetic hand systems [30], the real-time performance of the gesture recognition model determines the usability and user experience of the system. The real-time performance of the model can be assessed using both the attributes of prediction speed and response time, which indicate the model’s processing speed and response capacity to the input signal, respectively. Undoubtedly, quick response time and high prediction speed are critical in an EMG-controlled prosthetic hand system, allowing the users to control the hand accurately and sensitively in real time [31].

We compared the average response time (measured in seconds, s) and model prediction speed (measured in frames per second, fps) of different models, and the performance differences among the models are listed in Table 4. It is clear that the classical machine learning methods of KNN and SVM need long average response times (0.55 ± 0.05 s and 0.43 ± 0.08 s, respectively) and have low model prediction speeds (50 ± 5 fps and 60 ± 4 fps, respectively) due to their time-consuming feature engineering and complicated parameter tuning processes. The deep learning model of CNN, on the other hand, has the fastest average response time of 0.25 ± 0.07 s and the highest model prediction speed of 120 ± 6 fps, showcasing remarkable real-time performance. Adding LSTM to the CNN model somewhat increases the model’s average response time (0.34 ± 0.05 s), while reducing the model prediction speed to 85 ± 3 fps. This is determined by the characteristics of LSTM itself, namely, recursive computations, increased number of parameters, and training complexity. On the other hand, the average response time and prediction speed of the CNN model with the ECA mechanism added are 0.30 ± 0.06 s and 100 ± 5 fps, respectively. Although there is a slight sacrifice in real-time performance compared to the CNN model, the impact on the model’s overall performance is relatively small. This indicates that the ECA attention mechanism introduced in this study can indeed improve the model’s ability to focus on crucial information, so as to better balance model accuracy and real-time performance during task execution, and obtain satisfactory comprehensive performance.

Furthermore, it can be deduced from the data in Table 4 that our real-time control system for the prosthetic hand generates predictions at a high frequency, yielding a large number of classification results per second. However, since our proposed classifier does not achieve 100% accuracy, there are also instances of incorrect classifications in these results. To address this issue, this study employs the majority vote window technique to enhance the accuracy and stability of predictions.

**Figure 12:**
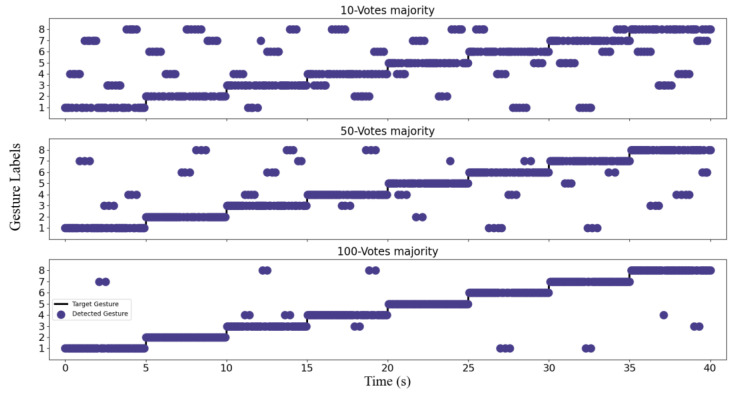
Output results of the majority window.

Figure 12 displays the results of gesture recognition within a 5-s timeframe as users perform each gesture sequentially with different majority voting window sizes (10, 50, and 100). Notably, the majority voting window is sliding, with the most recent prediction result added and the earliest prediction result removed at each step. This strategy ensures that the prediction results displayed within the voting window are always current and allow for real-time updates. The size of the majority voting window is a crucial parameter that affects real-time recognition performance. It can be seen from Figure 12 that compared with the prediction results when the window size is 10 and 50, the results obtained when the window size is set to 100 are very consistent with the true predictions, with no frequent fluctuations. These findings suggest that the majority voting window technique can effectively smooth out prediction outcomes, improve accuracy, suppress noise and fluctuations, and enhance the overall robustness of the model. Moreover, this technique is adaptable and flexible, making it highly suitable for real-time deep-learning models of sEMG signals on embedded devices.

## 4. Discussion

This research aims to improve the recognition performance and real-time capabilities of the sEMG prosthetic hand system by developing a sEMG signal processing framework based on the CNN-ECA model. Through comprehensive discussions in this section, we will delve into the methodology, experimental findings, and their significance in the field of EMG gesture recognition.

Deep learning models, with their fast training and inference speeds and powerful feature extraction capabilities, offer benefits in sEMG signal classification tasks. In this study, we use CNN as the base model and introduce the ECA module to improve the model’s attention to crucial features. The experimental results show the satisfactory performance of the CNN-ECA model in gesture recognition tasks. When compared to classical machine learning approaches and current mainstream deep learning models, our model outperforms them in terms of accuracy, recall, and F1 score. Importantly, our model distinguishes between multiple gesture categories better than existing deep learning models, enhancing the classification performance. This demonstrates that the introduction of the ECA mechanism has a positive impact on gesture recognition by increasing the model’s accuracy and focusing on essential information.

While the computational cost was proven to be competitive, it is crucial to delve into the complexity of the model. The low complexity of our model can be attributed to streamlined convolutional layers and the incorporation of the ECA module, which enhances the model’s attention mechanism without introducing unnecessary computational burden. Specifically engineered for efficiency, the ECA module strategically attends to relevant channels within the data, emphasizing crucial information without imposing excessive computational overhead. This ensures that the model becomes more adept at capturing key patterns and features without a proportional increase in computational requirements. This low complexity is advantageous for real-time applications, particularly in the context of prosthetic hand control, where quick and responsive processing is paramount. Notably, the achieved enhancement in accuracy comes without a substantial increase in computational complexity.

In addition, the real-time test with the prosthetic hand exemplifies the practical application of our research. It showcases that our model, with its efficient processing and real-time capabilities, can be effectively employed for controlling prosthetic hands through sEMG signals. The integration of the majority voting window technique allows the system to adapt to users’ muscle activity variations, ensuring the precise and reliable control of the prosthetic hand. A higher number of votes with a larger majority window enhances response stability but introduces a slight delay in recognizing rapid gesture changes, while a smaller number of votes with a smaller window size offers faster response times but may result in more prediction variability due to fewer samples. Moreover, through window adjustments, this study has ensured the real-time accuracy of prosthetic hand control.

Our research also has certain limitations that should be addressed in future work. The relatively small size of our experimental dataset may restrict the model’s generalization ability. Expanding the dataset will enhance the model’s robustness, particularly for individuals with limb amputations. Additionally, although our model has shown good real-time performance, there is room for further improvements in terms of algorithmic and hardware optimizations to enhance efficiency. Future work should explore more attention mechanisms and deep learning model architectures that consider both spatial and temporal aspects of sEMG signals. Developing processing algorithms and model structures tailored for individuals with limb amputations is of great importance. Integrating additional sensors and technologies, such as muscle tension sensors or neural sensing techniques, can further enhance the accuracy and robustness of gesture recognition systems. Moreover, optimizing the user experience and human–computer interaction for individuals with limb amputations will improve their quality of life and social participation. Collaboration and feedback from individuals with limb amputations and rehabilitation professionals will be essential in achieving these goals.

## 5. Conclusions

This study aims to improve the performance and real-time capabilities of the sEMG gesture recognition system by developing a muscle signal processing framework based on the CNN-ECA model. The application of preprocessing approaches including envelope extraction and sliding window segmentation can effectively extract useful information from surface sEMG data, reduce the influence of noise, and thus improve signal correlation and stability. The sEMG gesture recognition framework of CNN-ECA is developed based on the CNN architecture, where the introduction of the ECA module enabled the model to enhance the focus and capture ability of important features, thereby improving classification performance. Compared to classical machine learning approaches and the current mainstream deep learning models of CNN and CNN-LSTM, the CNN-ECA model we proposed shows significant improvements in the metrics of accuracy, recall, and F1 score. Moreover, the introduction of the ECA module does not significantly reduce the prediction speed of the model while improving the accuracy, implying that the ECA module simultaneously equips the EMG-controlled prosthetic hand system with the characteristics of accurate gesture detection and real-time control. In conclusion, the muscle signal processing framework based on the CNN-ECA model proposed in this study achieves favorable experimental results in the field of EMG gesture recognition. We believe that with further research and improvements, EMG gesture recognition systems will play a greater role in rehabilitation medicine, human–computer interaction, and virtual reality, significantly improving the quality of life and social participation of people with disabilities.

## Figures and Tables

**Table 1:** Performance comparison of ablation experiments.

Experimental Conditions	Accuracy	Loss
Conv1D	0.85	0.42
Conv2D	0.89	0.36
Conv2D+Attention	0.92	0.31
Our CNN-ECA	0.96	0.16

**Table 2:** The final optimal parameters.

Optimizer	Adam
Activation function	Relu
Learning rate	0.0001
Number of epochs	300
Batch size	128

**Table 3:** Recognition model comparison.

Model	Accuracy	Precision	Recall	F1 Score
KNN	0.71	0.69	0.72	0.70
SVM	0.80	0.77	0.73	0.75
LSTM	0.86	0.83	0.78	0.80
CNN-LSTM	0.93	0.92	0.87	0.89
Our CNN-ECA	0.96	0.95	0.92	0.94

**Table 4:** Comparison of the real-time performance of models.

Model	Average Response Time (s)	Prediction Speed (fps)
KNN	0.55 ± 0.05	50 ± 5
SVM	0.43 ± 0.08	60 ± 4
CNN	0.25 ± 0.07	120 ± 6
CNN-LSTM	0.34 ± 0.05	85 ± 3
Our CNN-ECA	0.30 ± 0.06	100 ± 5

## Data Availability

Data are unavailable due to privacy or ethical restrictions.
